# Trunk Detection in Complex Forest Environments Using a Lightweight YOLOv11-TrunkLight Algorithm

**DOI:** 10.3390/s25196170

**Published:** 2025-10-05

**Authors:** Siqi Zhang, Yubi Zheng, Rengui Bi, Yu Chen, Cong Chen, Xiaowen Tian, Bolin Liao

**Affiliations:** 1 College of Physics, Mechanical and Electrical Engineering, Jishou University, Jishou 416000, China; 18821983158@163.com (S.Z.); chenyu27183141@outlook.com (Y.C.); 2 College of Computer Science and Engineering, Jishou University, Jishou 416000, China; z26955@outlook.com; 3 Hunan Provincial Institute for Inspection and Testing of Special Equipment, Changsha 410000, China; birengui@jsu.edu.cn (R.B.); jy00427538@163.com (C.C.)

**Keywords:** dual-path feature decoupling, EffiDet, inspection robot, StarNet, YOLOv11 lightweight model

## Abstract

The autonomous navigation of inspection robots in complex forest environments heavily relies on accurate trunk detection. However, existing detection models struggle to achieve both high accuracy and real-time performance on resource-constrained edge devices. To address this challenge, this study proposes a lightweight algorithm named YOLOv11-TrunkLight. The core innovations of the algorithm include (1) a novel StarNet_Trunk backbone network, which replaces traditional residual connections with element-wise multiplication and incorporates depthwise separable convolutions, significantly reducing computational complexity while maintaining a large receptive field; (2) the C2DA deformable attention module, which effectively handles the geometric deformation of tree trunks through dynamic relative position bias encoding; and (3) the EffiDet detection head, which improves detection speed and reduces the number of parameters through dual-path feature decoupling and a dynamic anchor mechanism. Experimental results demonstrate that compared to the baseline YOLOv11 model, our method improves detection speed by 13.5%, reduces the number of parameters by 34.6%, and decreases computational load (FLOPs) by 39.7%, while the average precision (mAP) is only marginally reduced by 0.1%. These advancements make the algorithm particularly suitable for deployment on resource-constrained edge devices of inspection robots, providing reliable technical support for intelligent forestry management.

## 1. Introduction

In recent years, forest inspection automation has become the direction of future forestry development. Efficient inspection of forest resources is an important part of ecological protection, disaster early warning, and sustainable forestry management. Advanced technologies such as intelligent control, computer science, and sensor technology have been applied to the forestry sector, propelling modern forestry toward rapid development in automation, intelligence, and dynamic management [1,2,3]. As the main target object of the forest area, trunk recognition and location is an important part of the intelligent operation of the inspection robot [4].

At present, the global navigation satellite system (GNSS) has become the core positioning method for autonomous navigation of forest inspection robots with its global coverage characteristics. However, its positioning accuracy is notably lacking (error > 10 m) in dense forest environments, posing a serious threat to the safety and reliability of inspection under complex terrain [5]. While LiDAR equipped with VSLAM (Visual Simultaneous Localization and Mapping) offers a high-precision alternative, this scheme presents problems of high cost and substantial data processing requirements [6,7,8], making it less suitable for large-scale or cost-sensitive deployment. To bridge the gap between the insufficient accuracy of GNSS and the prohibitive cost of LiDAR, this study turns to visual recognition and positioning technology [9]. This approach offers a compelling balance, providing the necessary perceptual accuracy for trunk detection and localization at a fraction of the cost and computational overhead of LiDAR-based systems. Furthermore, considering the need for cost-efficiency and compactness of inspection robots, edge computing devices are considered the first choice for online detection tasks.

Recent studies have primarily focused on adapting lightweight YOLO architectures for trunk detection, yet these approaches exhibit notable limitations when deployed in complex, unstructured forest environments. These limitations can be categorized into several key challenges: First, while lightweight designs effectively reduce computational costs, they often struggle to maintain accuracy under challenging conditions. For instance, models incorporating modules like MobileNetV3 or GhostNet [10,11] achieve efficiency but may lack the robustness to handle significant occlusion and extreme lighting variations common in forests. Second, feature enhancement techniques, such as cross-layer fusion and attention mechanisms [12,13,14], improve feature representation but can introduce computational overhead that negates the benefits of lightweight backbones, making real-time performance on edge devices difficult to achieve. Furthermore, approaches relying on multi-modal data fusion (e.g., RGB-D) [15] enhance geometric perception but demand expensive sensors and complex data processing pipelines, which are impractical for low-cost, compact inspection robots. Finally, pruning-based methods [16] offer a path to compression but risk removing features critical for detecting irregular trunk shapes, leading to decreased generalization in diverse forest settings. Consequently, a significant gap remains in developing a trunk detection model that optimally balances lightweight architecture, high accuracy in complex scenes, and real-time inference efficiency—a balance crucial for the autonomous navigation of resource-constrained forest inspection robots.

This work is devised to mitigate these identified limitations. We posit that the paradox between model efficiency and accuracy can be resolved through a co-design of lightweight components and task-specific feature enhancement. Consequently, our proposed YOLOv11-TrunkLight algorithm introduces a novel lightweight backbone network for efficient feature extraction, a deformable attention module for handling geometric variations, and an efficient detection head for rapid inference, collectively ensuring robust performance under resource constraints. Furthermore, a purpose-built dataset mitigates the issue of data scarcity and lack of diversity. Our main contributions are threefold:

- To overcome the limitations of generic datasets and enhance model generalization for complex forest environments, we constructed a specialized dataset. This dataset was built by collecting images under various weather conditions and employing advanced data augmentation techniques, such as feature mapping transformation for background segmentation, cropping, and rotation. The processed trunk targets were then randomly scaled and superimposed onto diverse backgrounds to simulate the high variability and complexity of real-world forest scenes.
- Addressing the challenge of balancing lightweight design with feature representation power [10,11,12], we innovatively propose the StarNet_Trunk backbone network. This architecture replaces traditional residual connections with element-wise multiplication and integrates 3 × 3 depthwise separable convolutions. This design significantly reduces computational complexity and parameters while maintaining a large receptive field, thereby achieving efficient and robust trunk feature extraction without compromising the model’s capacity.
- To specifically enhance the model’s adaptability to the geometric deformation of trunks and complex backgrounds—a limitation not fully addressed by existing attention mechanisms [15,16] or multi-modal methods [14]—we designed the C2DA deformable attention module. It incorporates dynamic relative position bias encoding, allowing the model to dynamically adjust its receptive field to fit irregular trunk contours. Furthermore, to optimize the trade-off between detection accuracy and computational efficiency in the prediction head, we developed the dual-path EffiDet detection head. It improves detection speed and reduces parameters through feature decoupling and a dynamic anchor mechanism, making it highly suitable for edge deployment.

The remainder of this paper is organized as follows: Section 2 introduces the innovative aspects of the self-built dataset and improvements to YOLOv11 proposed in this paper. Section 3 describes the experimental preparations and analyzes the experimental results to demonstrate the performance of the improved model. Section 4 presents the main conclusions of this paper.

## 2. Materials and Methods

This section details the dataset construction process and the architecture of the proposed YOLOv11-TrunkLight model, including its key innovative components.

### 2.1. Dataset Construction

We describe the original image acquisition process, preprocessing, and annotation strategies, as well as the data augmentation techniques employed to enhance dataset diversity and robustness.

#### 2.1.1. Data Collection and Characterization

**Figure 1:**
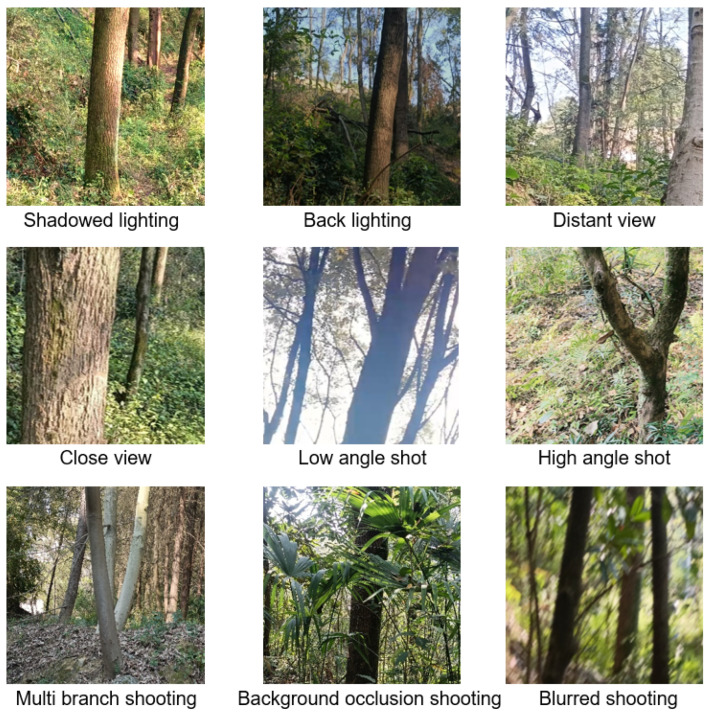
Photography conditions for different tree trunks.

The trunk image data for this study was collected in the Wuling Mountains region. This area features complex environmental conditions, presenting diverse trunk types and morphological characteristics. The collection process encompassed various meteorological scenarios, including sunny days, overcast conditions, and post-rain periods. Video recording and still photography were conducted using a camera (GoPro Hero 10) with a resolution of 3840 × 2160 pixels, capturing imagery under variable weather conditions at different times. The resulting video frames encompassed diverse photographic scenarios, including varying lighting conditions, shooting angles, obstructions, distances, and backlighting effects. Ultimately, 1000 images were obtained by integrating this material. Figure 1 displays a selection of tree trunk photographs collected from the Wuling Mountains region.

#### 2.1.2. Data Preprocessing and Annotation Strategy

**Figure 2:**
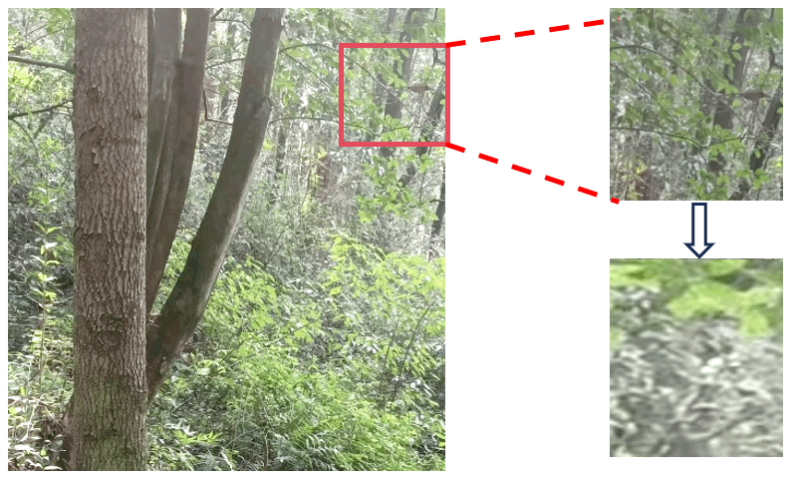
Image processing.

Therefore, considering that the camera’s field of view inevitably captures numerous distant tree trunks, and that the inspection robot’s actual operation only requires focusing on tree trunks near the working area for trajectory planning, this study employs neutral green to mask distant tree trunks (Figure 2). This operation aligns with robot navigation priorities by directing model attention toward nearby objects that hold practical significance for obstacle avoidance. Furthermore, from a learning perspective, distant tree trunks (characterized by low resolution and weak textures) exhibit statistically distinct visual feature distributions compared to nearby trunks. Using them as positive samples would introduce a “feature distribution bias,” confusing the feature representations of the model. By masking, we avoid the laborious task of annotating minute targets while proactively purifying learning objectives. This guides the model to establish robust associations between high-quality trunk features and detection tasks, thereby enhancing its generalization capabilities in real-world scenarios.

#### 2.1.3. Synthetic Data Augmentation

**Figure 3:**
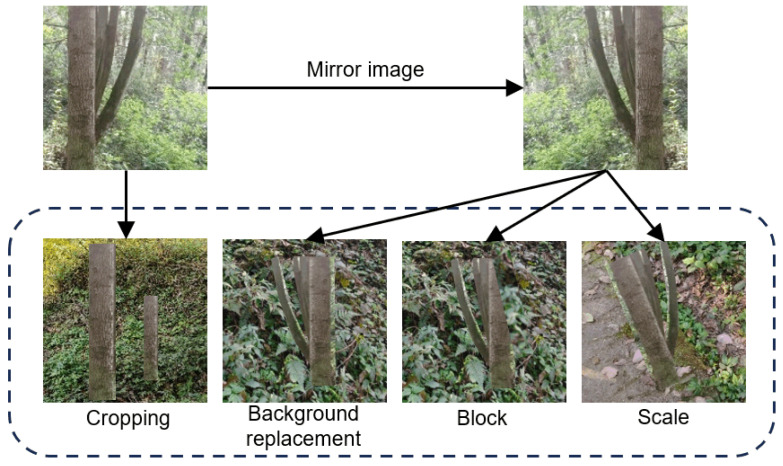
Illustration of the synthetic data augmentation procedure.

To fit the complex conditions of the forest, the dataset adopts the real collection dataset and the synthetic enhanced dataset construction scheme. The synthetic enhanced dataset can use Python and OpenCV feature map graphic transformation to cut, rotate, and replace the background segmentation and randomly scale the cropped and rotated core parts to the appropriate size; it is superimposed on different background images, and the position and size of the target image are randomly selected. Specifically, during augmentation, each trunk target was randomly rotated within a range of −30° to 30° to simulate varying viewpoints. The scaling factors were randomly sampled between 0.7 and 1.3 to create size variance. Finally, when pasting onto new backgrounds, the location was randomly chosen, allowing for partial occlusion near the image borders to improve model robustness (Figure 3). Following this processing, an enhanced dataset comprising 1000 images was obtained. Combined with the previously collected real dataset, this ultimately formed a comprehensive dataset containing 2000 images. The ratio of the real dataset to the synthetic enhancement dataset is 1:1. Finally, the self-built dataset is annotated according to the YOLO format to form a self-built dataset. According to the ratio of 9:1, it is divided into a training set and verification set.

### 2.2. Modeling

**Figure 4:**
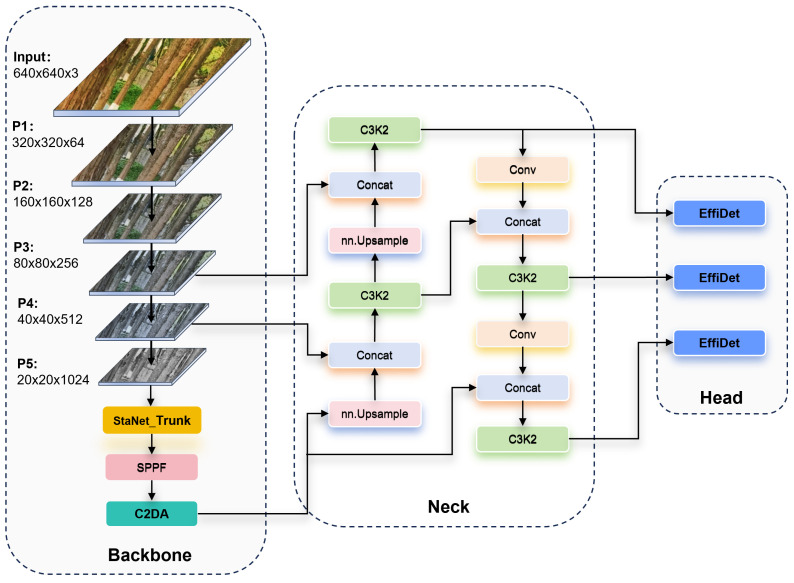
YOLOv11-TrunkLight lightweight trunk detection model.

Designed for trunk detection in complex forest areas, this study systematically optimizes the YOLOv11 network architecture and proposes a lightweight improved YOLOv11-TrunkLight model. As shown in Figure 4, the architecture of the original YOLOv11’s stacked C3K2 module suffers from high computational complexity. To address this issue, this study improved the StartNet_Trunk lightweight backbone network. This network adopts a four-stage hierarchical feature extraction architecture. The deep stage (third stage) allocates more blocks (3 blocks) to facilitate learning complex high-level semantic features—crucial for distinguishing tree trunks from complex backgrounds. The preceding and subsequent stages employ fewer blocks, respectively, efficiently handling low-level feature extraction and feature integration tasks to avoid parameter bloat. Thus, the number of blocks in each stage is optimized to [1,1,3,1] [17]. Each Block module is constructed by deeply separable convolution with residual connection, which significantly reduces the computational complexity of the model [18]. In particular, this study replaces the original C2PSA module with an innovative C2DA module that incorporates a deformable attention mechanism. This mechanism enables dynamic adjustment of the receptive field, significantly enhancing the model’s adaptability to geometric deformations of tree trunks [19]. The experimental results show that these improvements significantly reduce the computational burden of the model while ensuring the detection accuracy.

#### 2.2.1. StarNet_Trunk Module

The core advantage of StarNet module over YOLOv11 is its innovative lightweight design and efficient feature interaction mechanism [20]. StartNet_Trunk replaces the traditional additive residual connection with element-by-element multiplication, creating a gating mechanism that enhances feature selectivity. This nonlinear interaction method captures complex feature relationships more effectively than additive connections, particularly when combined with reduced channel dimensions from depthwise separable convolutions. The multiplicative operation acts as an attention-like mechanism without additional computational overhead, achieving a reduction in computational complexity from O(C^2^) for traditional attention mechanisms to O(C) for element-wise multiplication, where C represents the dimension of the channel. Meanwhile, the separable integrated 3 × 3 depthwise reduces the spatial complexity of the convolution from O(K^2^ × C^2^) to O(K^2^ × C), where K is the size of the kernel, allowing improved feature extraction accuracy while significantly reducing computational complexity and maintaining a large receptive field.

**Figure 5:**
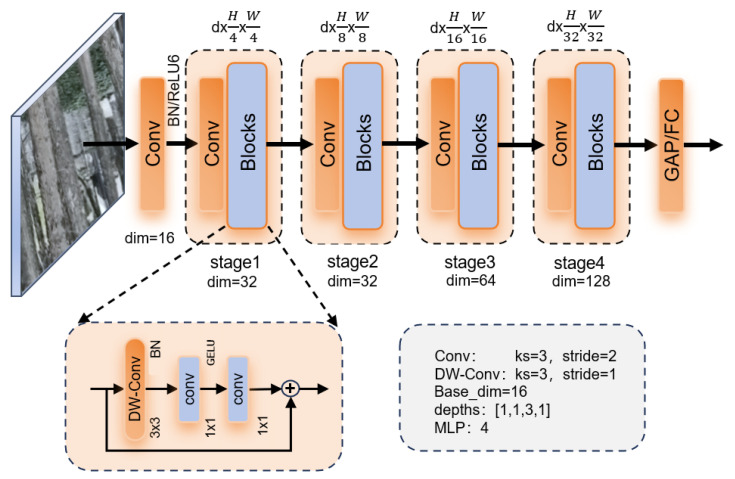
StartNet_Trunk module principle.

In terms of specific implementation, StartNet_Trunk achieves an attention-like adaptive feature selection capability through dual-channel feature transformation and dynamic multiplication fusion [21], without the need to introduce complex attention modules. Compared with the standard convolution stack and fixed addition interaction commonly used in YOLOV11, StartNet_Trunk can reduce the computational complexity of FLOPs, and the multiscale feature output is naturally adapted to the detection task. The element-wise multiplication inherently provides channel-wise feature modulation without requiring additional parameters, while depthwise separable convolutions maintain spatial feature extraction capability with significantly reduced computational cost. This synergistic design enables superior parameter efficiency through multiplicative gating, where the element-wise operation serves as an implicit attention mechanism that enhances feature discriminability in lightweight network architectures. The StartNet_Trunk Backbone Network adopts a multistage hierarchical feature extraction architecture, and its workflow can be divided into two core stages: initial feature extraction and multiscale feature fusion (Figure 5).

The input image first undergoes a 3 × 3 convolution with batch normalization and ReLU6 activation, which reduces spatial resolution while preserving high-frequency details. This operation reduces the amount of subsequent computation by decreasing the resolution while preserving high-frequency edge information. Subsequently, the feature map is sequentially passed through four stratification stages, each containing a downsampling module and a feature enhancement module. The downsampling module of the i-th stage expands the number of channels to a convolution of $C_{i}=base\;\_dim\times 2^{i}$, and its output is

(1) $$X_{\mathrm{down}\;}^{\left(i\right)}={\mathrm{ConvBN}}_{3\times 3}\left(X_{\mathrm{in}\;}^{\left(i\right)}\right).$$

This operation progressively reduces spatial dimensions to extract global contextual information while enhancing feature expressiveness through channel expansion. After downsampling, features are fed into a stacked architecture composed of multiple Block modules for deep processing. The four hierarchical stages contain [1,1,3,1] blocks, respectively. Compared to the traditional StarNet_s1 module, each Block in this design employs depthwise separable convolutions and integrates channel mixing with spatial attention mechanisms to optimize feature representation. The core innovation lies in reimagining traditional residual connections: we abandon the original additive operation Y=X+F(X) in favor of element-wise multiplication for feature fusion, expressed as Y=X⊙F(X). This multiplicative fusion mechanism inherently forms an efficient implicit attention model. It enables the network to dynamically and autonomously enhance important features or suppress redundant information across both the channel and spatial dimensions. This mechanism not only enhances the model’s ability to capture complex nonlinear relationships between features but also achieves a balance between performance and efficiency without introducing any additional parameters.

**Figure 6:**
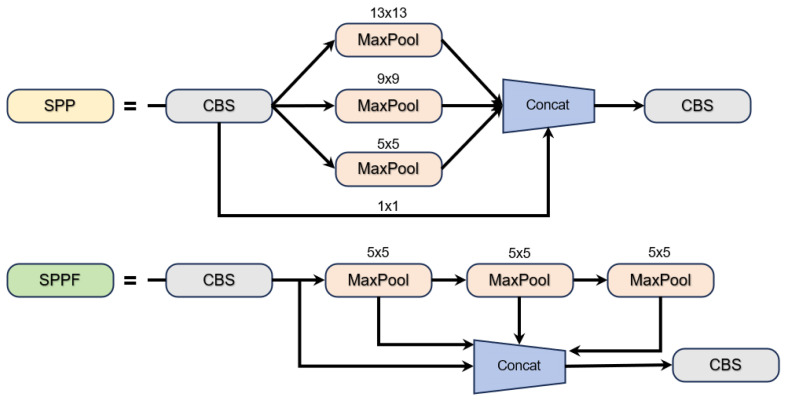
Schematic diagram of SPPF module.

Assuming the operation of a single block, the output of the th block can be recursively defined as

(2) $$X_{j}^{\left(i\right)}=F_{block}\left(X_{j-1}^{\left(i\right)},\theta_{mlp},drop\_path\right),$$

where $\theta_{mlp}$ represents the weight parameter of the Multilayer Perceptron (MLP), and “drop_path” is a stochastic depth regularization term, which is used to alleviate overfitting. The output of all “stage” is aggregated by joining jumps to form a multiscale feature pyramid $\left\{F_{1},F_{2},F_{3},F_{4}\right\}$, which provides complementary information of fine-grained local characteristics and coarse-grained semantic features for subsequent detection tasks [22]. Through the SPPF module, the three-level serial maximum pooling captures the multiscale context information, and the feature splicing is used to retain the original resolution information in order to aggregate the multiscale context information and improve the model’s ability to measure targets of different sizes (Figure 6). The specific mathematical formulas are as follows.

#### 2.2.2. C2DA Module

**Figure 7:**
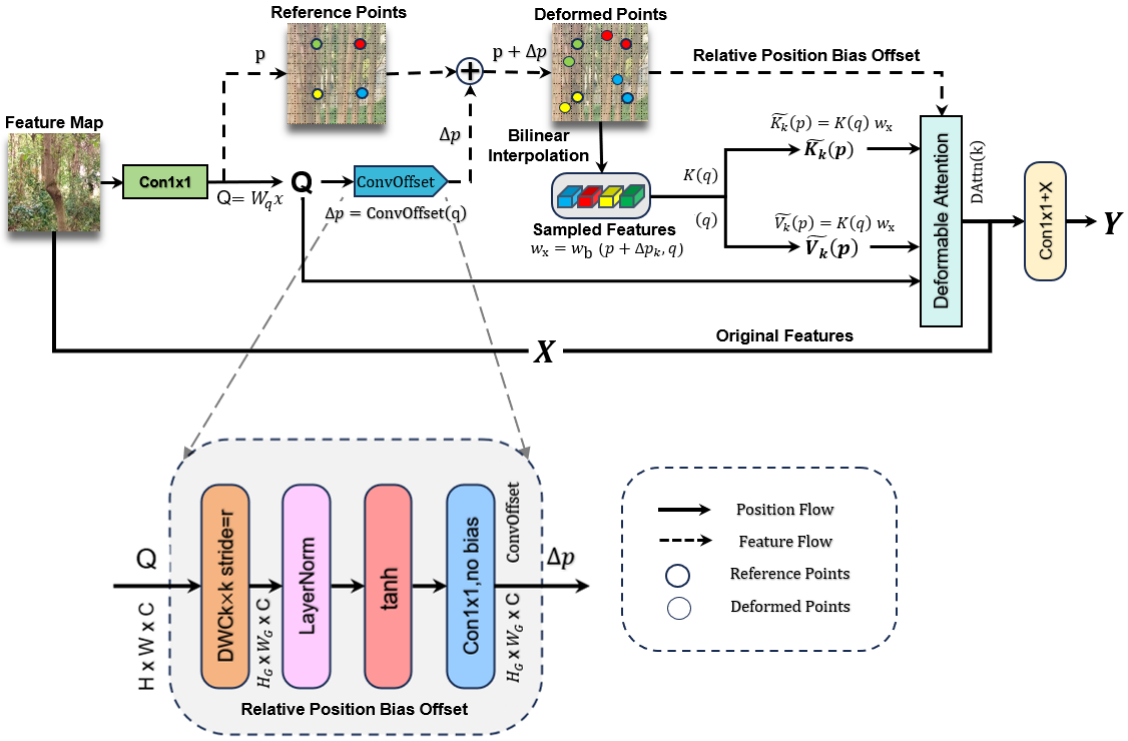
Schematic diagram of C2DA module.

The C2DA module inherits from the C2PSA architecture but achieves feature reconstruction by replacing the original PSABlock with DABlock [23]. Its core idea is to dynamically capture geometric deformation features of trunk targets through a deformable attention mechanism. The C2DA module reconstructs the C2PSA architecture through three key modifications: (1) Replacing the fixed PSABlock with a novel DABlock that implements a deformable attention mechanism. (2) Introducing a ConvOffset network to dynamically predict sampling offsets for irregular trunk geometries. (3) Implementing dynamic relative position bias encoding that learns spatial relationships in curved trunk structures, differing from C2PSA’s static position encoding. The core idea is to dynamically capture geometric deformation features of trunk targets through an enhanced deformable attention mechanism [24]. The workflow can be divided into three stages: feature triage, attention enhancement, and feature fusion (Figure 7).

First, the input feature map $x\in R^{B\times C\times H\times W}$ undergoes a 1 × 1 convolutional projection to obtain the query vector *Q*, the key vector *K*, and the value vector *V*:

(3) $$Q=W_{q}x,\;K=W_{k}x,\;V=W_{v}x,$$

where $W_{Q},W_{K},W_{V_{V}}$ is the learnable weight matrix. This operation compresses the feature channels to half the original number (controlled by parameter e = 0.5), reducing computational load while preserving key features. One branch retains the original features, while the other branch predicts a sampling offset for each query point *q*. Unlike standard attention mechanisms, C2DA does not use fixed key–value pairs. Instead, for each query point, a lightweight convolutional network predicts sampling offsets, enabling the model to focus on non-rectangular and deformed regions relevant to trunk shape. The other branch predicts *K* offsets (*K* typically ranges from 4 to 8; here, *K* is set to four) through this mechanism:

(4) $$\Delta p_{k}=\mathrm{ConvOffset}\left(q\right),$$

These predicted offsets are then used to dynamically sample a new set of keys and values from the original feature maps through bilinear interpolation, which ensures differentiability. The “ConvOffset” is a lightweight convolutional network in which the offset field is first passed through a deep convolutional network and then through a “tanh” activation function, which is used to control the magnitude of the deformation so that the model can better fit the complex data, and this operation is able to adapt to the irregular profile of the tree trunk [25]. The offset $\Delta p_{k}$ is obtained and then the feature maps (*K* and *V*) are dynamically sampled using a bilinear sampling function:

(5) $$\tilde{K_{k}}\left(p\right)=\;\sum \;K\left(q\right)\cdot w_{b}\left(p+\Delta p_{k},q\right),$$

(6) $$\tilde{V_{k}}\left(p\right)=\sum_{q\in N\left(p+\Delta p_{k}\right)}^{q\in N\left(p_{k}+\Delta p_{k}\right)}V\left(q\right)\cdot w_{b}\left(p+\Delta p_{k},q\right),$$

where N(p) denotes the neighborhood (usually of the four nearest pixels) and denotes the bilinear interpolation weight. The core deformable attention is computed by applying a standard attention mechanism (softmax over scaled dot-product) to the query and the newly sampled keys. A critical addition is the dynamic relative position bias, which explicitly encodes the spatial relationship between the query and its sampled points, further enhancing spatial awareness. DABlock performs deformable attention calculations. The core of DABlock lies in the computation of deformable attention, which computes the feature weights through the mechanism of multi-head attention:

(7) $$\mathrm{DAttn}\left(k\right)=\sum_{k=1}^{K}\mathrm{Softmax}\left(\frac{Q\tilde{K_{k}^{⊤}}}{\sqrt{d_{k}}}+\phi_{RPB}\left(\Delta p_{k}\right)\right)\tilde{V_{k}},$$

where $\phi_{RPB}$ is the position encoding function, and relative position bias is introduced to enhance spatial perception. The whole process significantly improves the robustness of trunk detection in complex backgrounds through dynamic sensory field adjustment and geometric feature enhancement [26]. Finally, the output from the deformable attention path is fused with the original features through a 1 × 1 convolution and a residual connection. This design ensures that important original information is retained while enhancing features with a dynamically attended context:

(8) $$y={\mathrm{Conv}}_{1\times 1}\left(\mathrm{Concat}\left[a,\mathrm{DAttn}\left(b\right)\right]\right)+x,$$

where *y* denotes the final output, and *x* denotes the original feature and denotes the branch that preserves the original feature. With this design, the localization accuracy of trunk detection is improved by local–global feature co-optimization while reducing the computational complexity. C2DA differs from the C2PSA position coding strategy by using a dynamic relative position bias, which learns the relationship between offset and position through “MLP”. This coding is adaptive to the spatial relationship of the local structure of the trunk (e.g., curved trunks), improves the modeling ability for irregular shapes, and enhances the robustness of the model to deformation:

(9) $$\phi_{RPB}\left(p_{i},p_{j}\right)=\mathrm{MLP}\left(\mathrm{Norm}\left(p_{i}-p_{j}+\Delta p_{ij}\right)\right),$$

where $p_{i}$ and $p_{j}$ denote the absolute coordinates of positions *i* and *j* on the feature map (normalized to the range [−1,1]), while $\Delta p_{ij}$ represents the predicted offset from position *i* to *j*, generated by the lightweight convolutional network described earlier. “Norm” refers to layer normalization, which stabilizes the training process and maps the positional relationship into an attention bias term. This dynamic relative position bias enables C2DA to adapt to the spatial relationship variations caused by trunk curvature and geometric irregularities, providing superior detection performance for non-cylindrical trunk shapes compared to traditional fixed attention mechanisms.

#### 2.2.3. EffiDett Module

**Figure 8:**
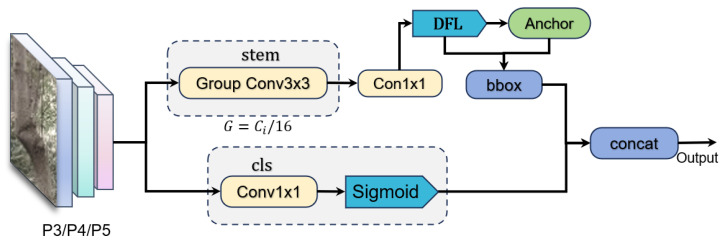
EffiDett module schematic.

The design of the EffiDet detector head adheres to three core objectives: improving the accuracy of regression for irregular tree trunk bounding boxes, maintaining robust classification confidence, and significantly reducing the number of parameters and computational latency. The core strategy to achieve these goals is the adoption of a dual-path feature decoupling scheme [27,28]. By separating the features used for regression and classification early in the detection head, each path can independently apply specialized optimization techniques—such as employing Distributed Focus Loss (DFL) for fine-grained localization and utilizing grouped convolutions to enhance feature processing efficiency—thereby achieving an optimal balance between accuracy and efficiency. Its core workflow comprises four stages: feature enhancement and decoupling, dual-task parallel prediction, dynamic anchor adaptation, and probability distribution decoding. Figure 8 illustrates the schematic diagram of the EffiDet module.

Input multiscale feature maps (P3/P4/P5) from Backbone with dimensions:

(10) $$P3:(F_{3}\in R^{B\times C_{3}\times H_{3}\times W_{3}}),$$

(11) $$P4:(F_{4}\in R^{B\times C_{4}\times H_{4}\times W_{4}}),$$

(12) $$P5:(F_{5}\in R^{B\times C_{5}\times H_{5}\times W_{5}}),$$

**Figure 9:**
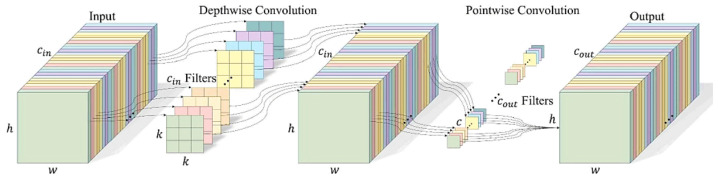
Deeply separable convolutional network.

After that, the feature maps of each scale go into the a-branch and b-branch, and the a-branch goes into the stem module, which consists of two layers of 3 × 3 grouped convolution, with each group of channels $G=\lfloor C_{i}/16\rfloor$, and each group is computed independently, and this operation reduces the amount of the original parameter to 1/16 of the standard parameter count, while utilizing the depth-separable convolution characteristic to preserve the multilayered spatial features to enhance the characterization of trunk texture and edges (Figure 9). Here, $c_{in}$ denotes the number of input channels, $c_{out}$ denotes the number of output channels, and *k* represents the size of the depth convolutional kernel.

This is followed by a two-branch prediction, with the a-branch predicting the regression offset and submitting a discrete distribution ‘4reg_max’ via lightweight convolution:

(13) $$Reg_{i}={Conv}_{1\times 1}\left(F_{i}^{\prime }\right)\in R^{B\times 64\times H_{i}\times W_{i}},$$

DFL (Distribution Focal Loss) decoding is then used to convert the discrete distribution into continuous offsets to optimize the small trunk localization:

(14) $$DFL=\sum_{k=0}^{15}Softmax\left(Reg_{i}\left[k\right]\right)\cdot k.$$

The complete DFL framework involves a loss function that guides the learning of this distribution. For a true target value $y_{t}$, the DFL loss concentrates on the two nearest integer bins *i* and *i* + 1, and is defined as

(15) $$DFL=-\left(\left(y_{t}-i\right)\cdot \log\left(P\left(i+1\right)\right)+\left(i+1-y_{t}\right)\cdot \log\left(P\left(i\right)\right)\right).$$

This forces the network to produce a sharp and confident distribution around the true value, leading to superior localization accuracy. Anchor point coordinates are then calculated by generating dynamic anchor boxes (adapting to input size changes):

(16) $${Anchor}_{i}=\left\{\left(x+0.5s,y+0.5s\right)|x,y\in Grid\left(H_{i},W_{i}\right)\right\},$$

where is the current layer step. Then the BBox is decoded and the final output of normalized center coordinates and width and height are determined to obtain the decoded bounding box:

(17) $${BBox}_{xywh}=\left({Anchor}_{i}\pm DFL\right)\times s,$$

the b-branch performs categorical prediction (Cls prediction) and outputs the category probabilities by lightweight convolution:

(18) $${Cls}_{i}=\sigma \left({Conv}_{1\times 1}\left(F_{i}^{\prime }\right)\right)\in R^{B\times N_{c}\times H_{i}\times W_{i}},$$

where σ is the activation function of the sigmoid. Finally, the multiscale prediction results are spliced to form a complete detection result. This process reduces the computation by grouping convolution, combines DFL to improve the small target detection ability, and is suitable for trunk detection tasks in complex environments:

(19) $$Output=Concat(\left[{BBox}_{3},{Cls}_{3}\right],\left[{BBox}_{4},{Cls}_{4}\right],\left[{BBox}_{5},{Cls}_{5}\right]),$$

where the multiscale outputs from three feature pyramid levels (P3, P4, P5) are concatenated to form the final detection result. Each level contributes both bounding box coordinates (bbox) and classification scores (Cls), enabling robust trunk detection across varying object scales.

## 3. Experimentation

This section outlines the experimental setup and evaluation metrics used to validate model performance and provides a detailed discussion of the results obtained through comparative and ablation studies.

### 3.1. Experimental Environment and Parameter Settings

We describe the hardware and software configurations used for training and testing, along with the specific hyperparameter values set to ensure reproducibility of results.

#### 3.1.1. Experimental Environment Configuration

In terms of experimental environment configuration, this study adopted high-performance hardware devices, including Nvidia RTX 4090gpu equipped with 24 GB of video memory, Core Intel i9-13900K CPU, and 64 GB of DDR5 memory to ensure the efficiency and stability of model training. The software environment is based on the Ubuntu 20.04 LTS operating system, using Python 3.8 programming language, coupled with the PYTORCH 2.0.1 deep learning framework and CUDA 11.8 acceleration library to maximize the computing power of the GPU. In addition, YOLOv11, the official implementation of Ultralytics, is adopted as the training framework to ensure the consistency and reproducibility of the model implementation. During the experiment, all the code runs in the Docker container to ensure the isolation of the environment and the reliability of the experimental results.

#### 3.1.2. Training Parameter Settings

In the training parameter setting, the input size of the model is fixed at 640 × 640 pixels to meet the needs of the target detection task. The batch size is set to 32 to avoid memory overflow while ensuring training efficiency. The optimization process adopts the ADAMW optimizer with an initial learning rate of 0.01 and is dynamically adjusted with a cosine annealing strategy to balance the convergence speed and generalization performance of the model. Meanwhile, a weight attenuation of 4 × $10^{-4}$ is set to prevent overfitting. A total of 150 training batches were conducted to ensure that the model fully learned the data features. In the aspect of data enhancement, the feature mapping graphics transform cuts, rotates and replaces the background segmentation, randomly scales the cropped and rotated core parts to the appropriate size, and superimposes them on different background images, to improve the robustness and generalization ability of the model. Some hyperparameter settings are shown in Table 1.

### 3.2. Evaluation Indicators and Standards

To comprehensively evaluate the performance of the proposed model, we adopt a set of metrics widely recognized in the field of object detection, which also serve as our evaluation criteria. The specific definitions are as follows:

#### Detection Accuracy Indicators

The $mAP_{50-95}$ is the core metric for comprehensively evaluating the model accuracy in the target detection task. $mAP_{50-95}$ is obtained by calculating the average precision (AP) at different IoU thresholds (0.5 to 0.95, step size 0.05) with a total of 10 IoU thresholds, and the AP for each threshold is calculated and then averaged. The formula is as follows:

(20) $$mAP_{50-95}=\frac{1}{10}\sum_{IoU=0.5}^{0.95}AP_{IoU},$$

This metric comprehensively evaluates the detection performance of the model under different IoU thresholds and is the gold standard for performance evaluation of target detection algorithms.

$mAP_{50}$ is the average precision when the IoU threshold is set to 0.5, calculated using the following formula:

(21) $$mAP_{50}=\frac{1}{N}\sum_{i=1}^{N}AP_{i},$$

where *N* is the number of categories and $AP_{i}$ is the average precision of the *i*-th category. This metric reflects the model’s ability to detect under relatively relaxed conditions.

Precision rate (*P*): the proportion of cases predicted to be positive that are actually positive, defined as

(22) $$P=\frac{TP}{TP+FP},$$

where TP is the number of true examples and FP is the number of false positive examples. High accuracy indicates that the model produces fewer false positives, which is important for forest inspection robots to avoid unnecessary obstacle avoidance maneuvers.

Recall (*R*): the proportion of actual positive examples that are correctly predicted, defined as

(23) $$R=\frac{TP}{TP+FN},$$

where FN is the number of false negative cases. High recall ensures that trunk targets are not missed, which is crucial for the navigation safety of inspection robots.

To evaluate the performance of the tree trunk detection model, this study employs the following core metrics: accuracy and recall measure detection precision, reflecting the model’s predictive accuracy and coverage of actual tree trunks, respectively; mAP comprehensively assesses detection quality; frame rate indicates the model’s real-time capability; while the number of parameters and the number of floating-point operations jointly evaluate model complexity and computational efficiency.

### 3.3. Comparative Analysis

We present a series of comparative experiments against state-of-the-art methods and between different components of our own model to demonstrate its effectiveness.

#### 3.3.1. Comparison with Mainstream Target Detection Algorithms

To validate the effectiveness of the proposed YOLOv11-TrunkLight, we provide a broad perspective encompassing diverse architectural philosophies and efficiency levels by carefully selecting models for comparison. We include mature two-stage detectors (Faster R-CNN, Cascade R-CNN) as high-precision benchmarks, popular one-stage detectors (SSD, RetinaNet, YOLOv8n, YOLOv11n) to achieve a balance between speed and accuracy, and models specifically designed for edge deployment (EfficientDet, YOLOv8n, YOLOv11n). This selection ensures our proposed method is evaluated against a robust and representative set of state-of-the-art alternatives. Table 2 presents the performance comparison results.

**Figure 10:**
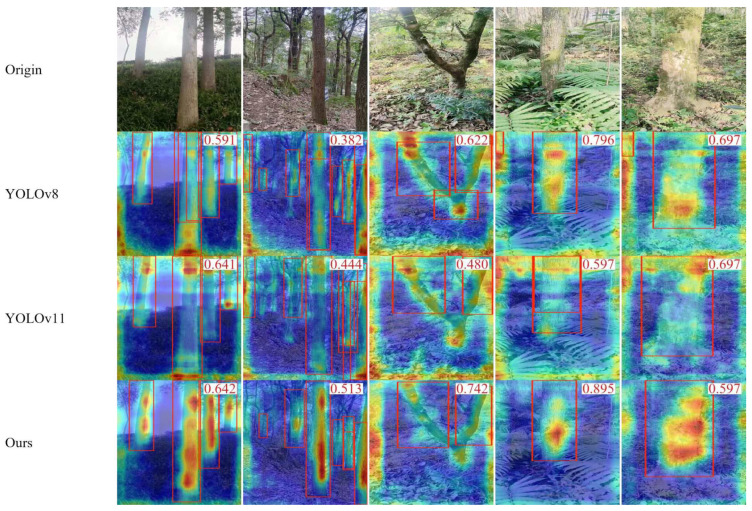
Comparison heatmap with YOLO series.

**Figure 11:**
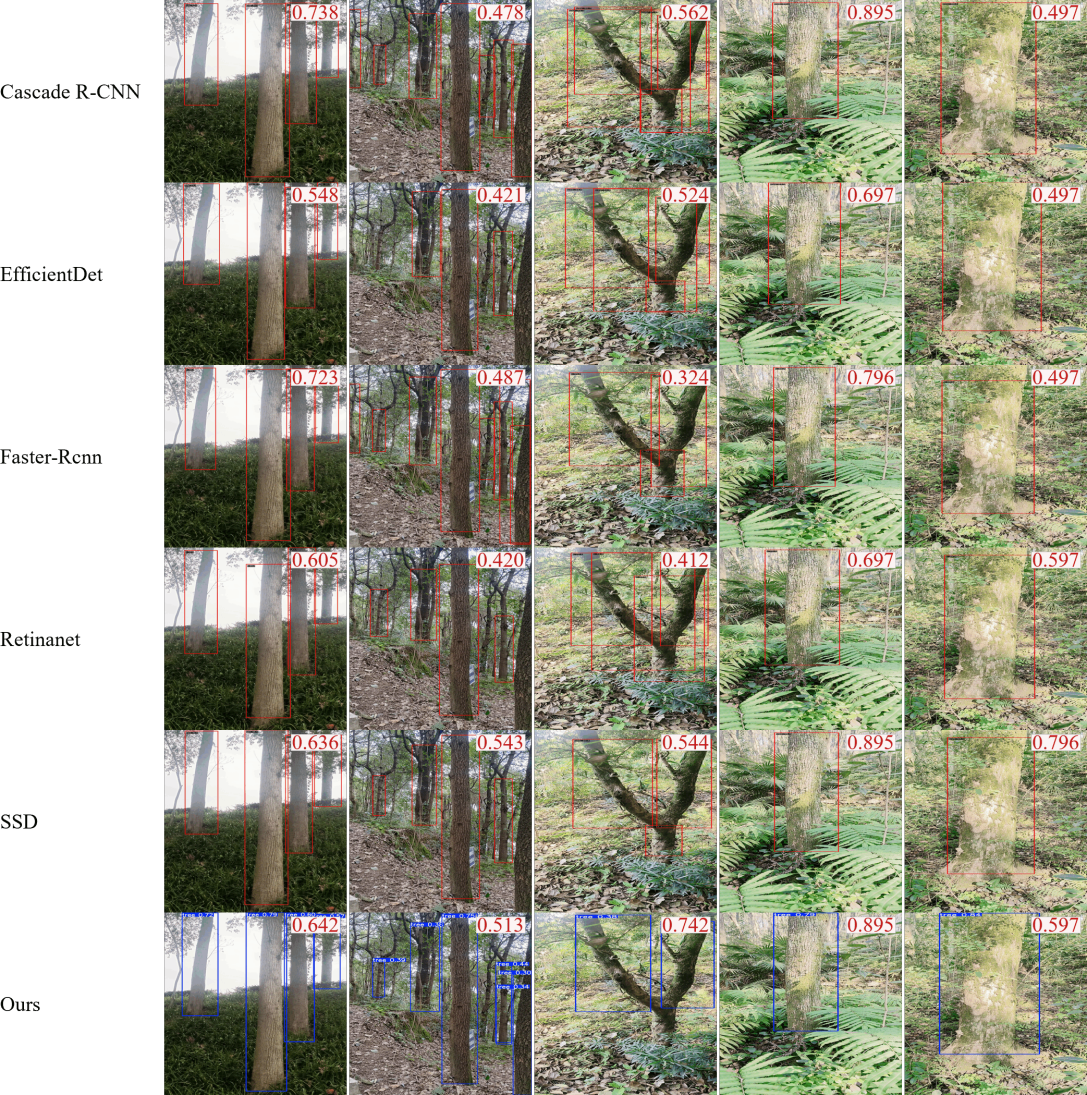
Comparative thermal diagram with non-YOLO series.

A comparison of the model of this study with its YOLO series visualization is shown in Figure 10 and with the non-YOLO series visualization in Figure 11.

Heatmap Analysis: Heatmaps (Figure 10 and Figure 11) provide an intuitive interpretation and comparison of spatial focus points (or “attention”) across different models. In these visualizations, warm colors (e.g., red) indicate image regions where the model places high reliance in its detection decisions, while cool colors (e.g., blue) represent areas largely ignored by the model. This enables us to move beyond quantitative metrics for qualitative comparisons, revealing how models of varying complexity or architectures approach the detection task. Experimental results show that while Cascade R-CNN achieves the highest mAP50–95 (56.90%), it requires significantly more computational resources. The baseline model in this study, YOLOv11-TrunkLight, achieves competitive performance with fewer parameters and lower computational complexity, making it more suitable for edge device deployment scenarios.

#### 3.3.2. Detection Head Comparison Experiment

In this study, different detection head architectures were evaluated to validate the effectiveness of the proposed EffiDet detection head. Table 3 summarizes the comparison results.

The EffiDet detection head proposed in this study achieves competitive accuracy while maintaining the lowest computational cost (2.312139 M parameters, 5.1 G FLOPs). Notably, EffiDet has a significantly reduced computational effort compared to other lightweight detection heads, which is a key requirement for autonomous navigation applications.

#### 3.3.3. Backbone Network Assessment

This study conducted extensive experiments on different backbone network architectures for the trunk detection task. Table 4 demonstrates the comparison results.

The results show that the StarNet_Trunk backbone network proposed in this study exhibits the most efficient parameter utilization (1.942563 M parameter counts, 5.0 G FLOPs) while maintaining the engagement precision (92.20% mAP50). And the higher recall of StarNet_Trunk (86.30%) indicates its effectiveness in detecting trunk instances, which is crucial for navigation safety.

### 3.4. Ablation Experiments

This part systematically dissects the contribution of each proposed component in our model through controlled experiments.

#### 3.4.1. Component Contribution Analysis

To validate the contribution of each proposed component, comprehensive ablation experiments were conducted in this study. Table 5 demonstrates the performance and efficiency analysis and Table 6 shows the accuracy analysis.

As shown in Table 5 and Table 6, our YOLOv11-TrunkLight model achieves a significant reduction in parameters (34.6%) and computational load (FLOPs, 39.7%), while also substantially improving inference speed (FPS, 13.5%)—core objectives of this study for edge deployment. This efficiency gain comes with a slight decrease in the stringent mAP50–95 metric (from 54.9% to 53.3%). We consider this trade-off positive for two reasons: First, the mAP50–95 decline is minimal (1.6 percentage points, a relative decrease of 2.9%), while the efficiency improvement is substantial. Second, and more critically, the model maintains strong competitiveness on the more practically valuable mAP50 metric at 92.9%, indicating its core capability to accurately locate tree trunks under standard conditions remains largely preserved. Thus, the model successfully achieves its design goal: prioritizing substantial efficiency gains for real-time operation on edge devices while preserving the high accuracy required for reliable forest navigation.

#### 3.4.2. Component Analysis

Impact of C2DA module: the deformable attention mechanism in C2DA specifically addresses geometric deformation challenges in trunk detection. The dynamic offset prediction enables adaptive sampling from irregularly shaped trunk regions, resulting in an improved recall rate from 80.10% to 86.20%, particularly effective for curved and partially occluded trunks compared to C2PSA’s fixed attention patterns. The average precision is improved from 93.00% to 94.40%, with computational overhead remaining minimal.

EffiDet Detection Head Contribution: replacing the original detection head with EffiDet reduces the computational complexity from 6.3 G to 5.1 G while maintaining competitive accuracy. The inference speed is improved from 218.87 f/s to 276.52 f/s, demonstrating the efficiency gained through grouped convolution and dynamic anchor frame mechanism.

StarNet_Trunk backbone network: the lightweight StarNet_Trunk backbone network significantly reduces the model parameters from 2.329 M to 1.689 M (27.5% reduction) and FLOPs from 5.1 G to 3.8 G (25.5% reduction). However, the small change in accuracy (53.20% to 53.30% mAP50–95) indicates that the recognition accuracy of the model was effectively ensured during the model compression process. Synergy: the complete YOLOv11-TrunkLight model achieves an optimal balance between accuracy and efficiency. With a configuration of 1.689 M parameters and 3.8 G FLOPs, it maintains 92.90% mAP50 and 53.30% mAP50–95 while achieving 275.37 FPS, demonstrating superior deployment feasibility on edge devices.

## 4. Conclusions

This chapter summarizes the key findings of this research, reiterates the core contributions of the proposed YOLOv11-TrunkLight algorithm, and explores its broader implications. It also outlines the limitations of the current work and potential directions for future research.

### 4.1. Research Findings Overview

This study proposes the YOLOv11-TrunkLight lightweight object detection algorithm, specifically designed for autonomous navigation of forest inspection robots in complex environments. Experimental results demonstrate that while maintaining high detection accuracy (mAP decreased by only 0.1%), this model achieves significant reductions in parameter count (34.6% reduction) and computational load (39.7% reduction), alongside a 13.5% improvement in inference speed.

### 4.2. Key Innovations and Advantages

Exceptional performance stems from three core innovations: (1) The StarNet_Trunk backbone reduces computational complexity through element-wise multiplication and depth-separable convolutions. (2) The C2DA module’s deformable attention mechanism enhances robustness against tree trunk geometric deformations. (3) The EffiDet detector head optimizes the speed–accuracy trade-off via dual-path feature decoupling. This holistic lightweight design grants the model decisive advantages in edge deployment compared to heavier, computationally intensive alternatives.

### 4.3. Implications and Future Deployment

Application Prospects and Future Deployment: The YOLOv11-TrunkLight algorithm is particularly suited for resource-constrained edge computing device deployment. It provides a reliable and efficient technical solution for high-precision tree trunk detection, strongly supporting the development of intelligent forestry management and autonomous robotic inspection systems.

## 5. Limitations and Future Work

This chapter candidly addresses the limitations and shortcomings of this study and proposes specific research directions and improvement strategies.

### 5.1. Model Limitations

The primary limitation of this study lies in its geographical specificity: the model’s generalization capability to unknown forest environments with different dominant tree species remains unvalidated. Additionally, the system faces challenges in demonstrating robustness under all weather conditions (e.g., snowfall, haze) and in handling dynamic scenes.

### 5.2. Future Research Directions

Consequently, future research will focus on three directions: (1) Enhancing model generalization through large-scale cross-regional data collection and domain adaptation techniques. (2) Integrating complementary sensor modalities such as RGB camera data and thermal imaging to improve robustness. (3) Extending static detection models to a spatiotemporal framework to enable temporal tracking of tree trunks, thereby providing more reliable navigation support in dynamic environments. 

## Figures and Tables

**Table 1:** Parameter settings.

Parameters	Numbers/Types
Image size/(pixel × pixel)	640 × 640
Training batch	150
Data loading thread	8
Initial learning rate	0.01
Weight decay factor	0.0004

**Table 2:** Performance comparison with mainstream target detection algorithms.

Model	mAP50–95 (%)	mAP50 (%)	P (%)	R (%)	Params (M)	FLOPs (G)
Easter-Renn	54.70	94.80	92.30	87.60	60.34	247.81
SSD	52.20	91.40	91.00	82.70	24.38	87.541
Retinanet	50.90	92.80	96.40	81.20	36.33	177.152
EfficientDet	52.00	93.70	87.50	84.90	18.33	79.786
Yolov8n	53.60	92.80	87.10	85.90	2.87	8.1
Yolov11n	54.90	93.00	93.90	80.10	2.58	6.3
Cascade R-CNN	56.90	93.30	91.10	85.40	88.14	276.18
Ours	53.30	92.90	90.80	85.40	1.68	3.8

**Table 3:** Performance comparison of different sensor head architectures in trunk detection tasks.

Model	mAP50–95 (%)	mAP50 (%)	P (%)	R (%)	Params (M)	FLOPs (G)
Aux	54.80	94.10	84.00	89.20	2.582347	6.3
Atthead	44.20	83.20	82.30	74.50	2.596011	6.5
MAN	53.80	93.90	86.00	87.90	3.774299	8.4
SRFD	54.40	93.00	88.70	85.10	2.554027	7.6
SEAMHead	53.70	93.00	87.70	84.70	2.490571	5.8
WFU	54.80	94.00	86.30	87.80	3.600075	8.0
EffiDet	54.00	93.20	89.70	84.50	2.312139	5.1

**Table 4:** Performance comparison of different backbone networks in trunk detection tasks.

Model	mAP50–95 (%)	mAP50 (%)	P (%)	R (%)	Params (M)	FLOPs (G)
fasternet	54.20	93.30	92.70	81.20	3.901959	9.2
timm	53.60	92.60	93.60	80.00	13.056003	33.6
efficientViT	55.20	92.50	88.80	82.30	3.738051	7.9
SPDConv	54.60	92.90	87.10	85.90	4.586827	11.3
swintransformer	54.10	92.50	89.50	85.40	29.715709	77.6
LDConv	53.70	92.70	90.40	83.20	2.166127	5.4
Starnet_Trunk	52.20	92.20	86.10	86.30	1.942563	5.0

**Table 5:** Comprehensive analysis of the impact on component performance and efficiency 1.

Baseine	C2DA	EffiDet	StarNet_Trunk	Params (M)	FLOPs (G)	mAP50 (%)	FPS
√				2,582,347	6.3	93.00	242.58
√	√			2,599,307	6.3	94.40	218.87
√	√	√		2,329,099	5.1	93.30	276.52
√	√	√	√	1,689,315	3.8	92.90	275.37

**Table 6:** Comprehensive analysis of the impact on component performance and efficiency 2.

Baseline	C2DA	EffiDet	StarNet_Trunk	P (%)	R (%)	mAP50–95 (%)
√				93.90	80.10	54.90
√	√			89.00	86.20	54.40
√	√	√		89.00	83.00	53.20
√	√	√	√	90.80	85.40	53.30

## Data Availability

The original contributions presented in this study are included in the article. Further inquiries can be directed to the corresponding author(s).
